# Alpha4 beta7 integrin controls Th17 cell trafficking in the spinal cord leptomeninges during experimental autoimmune encephalomyelitis

**DOI:** 10.3389/fimmu.2023.1071553

**Published:** 2023-04-18

**Authors:** Barbara Rossi, Silvia Dusi, Gabriele Angelini, Alessandro Bani, Nicola Lopez, Vittorina Della Bianca, Enrica Caterina Pietronigro, Elena Zenaro, Carlotta Zocco, Gabriela Constantin

**Affiliations:** ^1^ Department of Medicine, University of Verona, Verona, Italy; ^2^ The Center for Biomedical Computing (CBMC), University of Verona, Verona, Italy

**Keywords:** alpha4 beta7 integrin, Th1 cells, Th17 cells, leptomeninges, intravital microscopy, experimental autoimmune encephalomyelitis

## Abstract

Th1 and Th17 cell migration into the central nervous system (CNS) is a fundamental process in the pathogenesis of experimental autoimmune encephalomyelitis (EAE), the animal model of multiple sclerosis (MS). Particularly, leptomeningeal vessels of the subarachnoid space (SAS) constitute a central route for T cell entry into the CNS during EAE. Once migrated into the SAS, T cells show an active motility behavior, which is a prerequisite for cell-cell communication, *in situ* reactivation and neuroinflammation. However, the molecular mechanisms selectively controlling Th1 and Th17 cell trafficking in the inflamed leptomeninges are not well understood. By using epifluorescence intravital microscopy, we obtained results showing that myelin-specific Th1 and Th17 cells have different intravascular adhesion capacity depending on the disease phase, with Th17 cells being more adhesive at disease peak. Inhibition of αLβ2 integrin selectively blocked Th1 cell adhesion, but had no effect on Th17 rolling and arrest capacity during all disease phases, suggesting that distinct adhesion mechanisms control the migration of key T cell populations involved in EAE induction. Blockade of α4 integrins affected myelin-specific Th1 cell rolling and arrest, but only selectively altered intravascular arrest of Th17 cells. Notably, selective α4β7 integrin blockade inhibited Th17 cell arrest without interfering with intravascular Th1 cell adhesion, suggesting that α4β7 integrin is predominantly involved in Th17 cell migration into the inflamed leptomeninges in EAE mice. Two-photon microscopy experiments showed that blockade of α4 integrin chain or α4β7 integrin selectively inhibited the locomotion of extravasated antigen-specific Th17 cells in the SAS, but had no effect on Th1 cell intratissue dynamics, further pointing to α4β7 integrin as key molecule in Th17 cell trafficking during EAE development. Finally, therapeutic inhibition of α4β7 integrin at disease onset by intrathecal injection of a blocking antibody attenuated clinical severity and reduced neuroinflammation, further demonstrating a crucial role for α4β7 integrin in driving Th17 cell-mediated disease pathogenesis. Altogether, our data suggest that a better knowledge of the molecular mechanisms controlling myelin-specific Th1 and Th17 cell trafficking during EAE delevopment may help to identify new therapeutic strategies for CNS inflammatory and demyelinating diseases.

## Introduction

T cells constantly circulate in the bloodstream searching for the right signals to migrate into lymphoid or non-lymphoid organs. Several steps are required for T cells to arrive in a target site. Firstly, they need to cross the vascular wall of the post-capillary venules, a process that is governed by a sequence of events including: 1) tethering and rolling, which are mediated by the interactions between selectins and mucins, and/or between integrins and adhesion molecules from the immunoglobulin superfamily; 2) activation induced by chemoattractants, leading to integrin activation; 3) arrest and intravascular crawling mediated by integrins and their endothelial ligands; and 4) diapedesis or transmigration, which represents a complex step mediated by receptors such as endothelial cadherins and junctional adhesion molecules (1, 2). After extravasation, T cells may perform long-spread migration and communicate with other cells by chemical signaling and cell-cell physical interactions. The dynamic behavior of T cells is crucial for antigen recognition, proliferation and survival, leading to immune response development (3, 4). Indeed, *in vivo* high motility capability of T cells is an essential feature for an efficient antigen scanning in lymphoid organs or inflamed target tissues (5–7).

Multiple sclerosis (MS) and its animal model, experimental autoimmune encephalomyelitis (EAE), are considered autoimmune diseases mediated by myelin-specific encephalitogenic T lymphocytes, which migrate into the central nervous system (CNS) and become reactivated by local antigen presenting cells (APCs) (8, 9). Using a transfer model of EAE and two-photon laser scanning microscopy (TPLSM), previous studies demonstrated that pioneer T cells preferentially enter the CNS migrating across pial vessels within the meningeal space. Here, T cells assume a “migratory phenotype” leading to the subsequent invasion of spinal cord parenchyma, which represents a fundamental step for clinical EAE development (10). In addition, epifluorescence intravital microscopy (EIVM) studies performed by our laboratory demonstrated that activated T cell adhesion in inflamed CNS pial vessels is controlled by commonly used leukocyte adhesion molecules, such as selectins, mucins, chemokines, integrins and members of the immunoglobulin superfamily (11–16). Currently, it is well established that α4 integrins are key players in mediating tethering and rolling as well as firm adhesion of encephalitogenic T cells in inflamed brain pial venules (14). However, the individual contributions of the two related α4 integrins, α4β1 (or very late antigen-4 (VLA-4)) and α4β7 (also known as lymphocyte Peyer’s patch adhesion molecule (LPAM)), in intravascular T cell rolling and firm adhesion (arrest) during EAE is not well understood. Also, how adhesion molecules control the motility behavior of infiltrating encephalitogenic T cells inside the subarachnoid space (SAS) during early EAE and the subsequent penetration of T cells into the CNS parenchyma were not well investigated.

A role for lymphocyte function-associated antigen-1 (LFA-1 or αLβ2) in the amoeboid encephalitogenic T cell migration in the SAS during EAE was recently established, but whether α4 integrins also contribute to T cell motility behavior is unclear (17). In this work, we investigated the roles of α4 integrins in the migration of Th1 and Th17 cells, two cell populations involved in the induction of autoimmunity and neuroinflammation during EAE (18). We first studied the roles of α4β1 and α4β7 integrins in Th1 and Th17 cell intravascular adhesion by using EIVM in the spinal cord leptomeningeal venules at different disease phases in mice immunized with the myelin oligodendrocyte glycoprotein (MOG)_35-55_ peptide. Then, we performed TPLSM studies to determine the role of α4 integrins in the spinal cord SAS motility behavior of adoptively transferred Th1 and Th17 cells. Our data show that, whereas VLA-4 has a central role in Th1 cell migration, α4β7 integrin is selectively involved in Th17 cell trafficking in the leptomeninges during EAE, suggesting that understanding integrin-dependent T cell dynamics may lead to the therapeutic modulation of immune responses during CNS inflammatory and autoimmune diseases (19).

## Materials and methods

### Study approval

The studies involving mice were authorized by the Italian Ministry of Health, Department of Veterinary Public Health, Nutrition and Food Safety, Directorate General of Animal Health and Veterinary Medicine, as required by the Italian legislation (D.Lgs 26/2014, application of European Directive 2010/63/EU). Protocol numbers 33588, 30969 and 96/2023-PR were used for the *in vivo* studies in this manuscript.

### Mice

C57BL/6J wild-type (WT) mice and C57BL/6-Tg (Tcra2D2,Tcrb2D2)1Kuch/J (2D2 TCR) transgenic mice carrying a T cell receptor specific for the MOG_35-55_ peptide (20) were obtained from The Jackson Laboratory. All efforts were made to minimize the number of animals used and their suffering during the experimental procedure. All animal experiments were supervised by the local Institutional Animal Care Committee (OPBA) of the University of Verona and were conducted according to current European Community rules and approved protocols as mentioned above.

### Production of MOG_35-55_-specific Th1 and Th17 cells

Spleens were collected from 2D2 TCR transgenic mice, and a single-cell suspension was prepared and cultured as previously described (17). Briefly, splenocytes were cultured in the presence of 20 µg/ml MOG_35-55_ peptide (GenScript Corporation). Th1 polarization was induced by *in vitro* stimulation with 1 ng/ml IL-12 (R&D Systems) and 10 µg/ml anti-IL-4 antibody (clone 11B11, hybridoma kindly provided by E. C. Butcher, Stanford University), while Th17 polarization was induced by using 5 ng/ml TGF-β, 20 ng/ml IL-6 and 2 ng/ml IL-23 (all from Miltenyi Biotech or R&D Systems), as well as 10 µg/ml anti-IL-4 antibody (as above) and 10 µg/m anti-IFNγ antibody (clone HB170, R&D Systems). After 4 days in culture, Th1 and Th17 cells were supplemented with IL-2 (20 U/ml) or IL-7 (10 ng/ml) respectively for other 72 h. MOG_35-55_-specific Th1 and Th17 cells were then isolated using a Ficoll-Paque density gradient (GE Healthcare Life Sciences) and re-stimulated for 3 days in the presence of irradiated splenocytes as APCs (APC:T cell ratio = 5:1) with the same protocol used for the first stimulation. Th1 and Th17 cells were then isolated as described above and supplemented for one further day with IL-2 or IL-7, respectively. Th1 and Th17 polarization was confirmed by flow cytometry (**Supplementary Figures 1A, B**) as previously described (17).

### Isolation of Th1 and Th17 cells from EAE induced mice tissues

Endogenous leukocytes were collected from the blood and spinal cord of C57BL/6 MOG_35-55_-immunized EAE mice at disease peak. Exogenous *in vitro* differentiated CMAC-labelled Th1 or Th17 cells were injected in EAE mice at disease onset and collected from the spinal cord 48 h later. Blood was harvested from retro-orbital plexus of anesthetized mice before perfusion, and leukocytes separated from red cells by dextran sedimentation followed by hypotonic lysis. Mice were then immediately perfused through the left cardiac ventricle by injecting cold phosphate buffered saline (PBS) supplemented with 1mM Ca2+/Mg2+. Spinal cords were collected, homogenized using GentleMACS Octo Dissociator (Miltenyi Biotec), enzymatically digested with 1 mg/ml collagenase (Sigma Aldrich) and 40 U/ml DNase (Thermo Fisher Scientific) for 45min at 37°C and leukocytes isolated from the cell suspension by Percoll gradient centrifugation as previously described (17).

### Flow cytometry

*In vitro* differentiated Th1 and Th17 cells were stimulated for 12 h with 50 ng/ml phorbol 12-myristate 13-acetate (PMA), 1μg/ml ionomycin, and 10μg/ml brefeldin A for cytokine production assessment. Cells were fixed and permeabilized and labelled with FITC-conjugated anti-IFNγ and PE-conjugated anti IL-17 antibodies (Biolegend). *In vitro* differentiated Th1 and Th17 cells were also stained with the following rat anti-mouse unconjugated monoclonal antibodies for the evaluation of adhesion molecule expression: anti-CD49d (clone PS-2), anti-LPAM-1 (clone DATK32), anti-CD44 (clone MJ64), anti- CD11a/CD18 (clone TIB213), anti-L-selectin (clone MEL-14) and anti-PSGL-1 (clone 2PH1). A PE Goat anti-rat IgG was used as secondary antibody (Biolegend). For *ex vivo* studies, in exogenous Th1 and Th17 cells, the mean fluorescence intensity (MFI) of α4β7 expression (FITC- conjugated anti-α4β7 antibody; BidScientific) was calculated on live CMAC+ infiltrating cells. Endogenous Th1 and Th17 cells were identified by chemokine receptors profile and assessed for α4β7 expression by using the following antibody panel: VioBlue-conjugated anti-CD45 (Miltenyi Biotec), VioGreen-conjugated anti-CD45 (Miltenyi Biotec), FITC- conjugated anti-α4β7 (BidScientific), PE-conjugated anti-CCR6 (Biolegend), PE-Cy7-conjugated anti-CXCR3 (Biolegend), APC-conjugated anti-CCR4 (Biolegend). Cell viability was assessed by 7-AAD labelling (Biolegend) for freshly isolated cells or by Viobility 405/520 Fixable Dye (Miltenyi Biotec) for fixed cells. Cells were acquired by a MACSQuant Analyzer (Miltenyi Biotec) and the analysis was performed with FlowJo software.

### Surgical procedures for intravital microscopy

Mice were anesthetized and prepared for surgery to expose the lumbar column over L1-L4 leaving an intact dura mater as previously described (17). Mice were positioned on the stabilizing device and maintained at 37°C. Local administration of few drops of artificial CSF on surgically exposed spinal cord (Cold Spring Harb Protoc; 2011; doi:10.1101/pdb.rec065730) allowed the direct immersion of the microscope objectives.

### Epifluorescence intravital microscopy (EIVM)

Th1 and Th17 cells were labeled for 5 min at 37°C with either green 5-chloromethylfluorescein diacetate (CMFDA) or orange 5-(and-6)-(((chloromethyl)benzoyl)amino)tetramethylrhodamine (CMTMR) (both from Molecular Probes) (11). 3 × 10^7^ of differentially labelled Th1 and Th17 cells were then intravenously injected immediately before imaging into MOG_35-55_-immunized EAE recipient mice at different disease phases. In some experiments, cells were preincubated with blocking antibodies and compared to untreated control T cells. Particularly, Th1 and Th17 cells were incubated with 100 μg/ml of anti-α4 integrins (clone PS/2, rat anti-mouse) or anti-α4β7 integrin (clone DATK32, rat anti-mouse), anti-LFA-1 (clone TIB213, rat anti-mouse) for 5 min at 37°C and then injected with a supplement of up to 100 µg of blocking antibody (11). Mice were placed under a water immersion objective with a long focal distance (Olympus Achroplan, focal distance 3.3 mm, NA 0.5) of an upright Olympus BX50WI microscope. A Sony SSM-125CE monitor linked to a VE-1000 SIT analog monochromatic silicon-intensified target camera (Dage-MTI) allowed images visualization while an HDV DN-400 recorder (Datavideo Technologies Co.) was used for digitalization and storage of real-time movies (16) (**Supplementary Movie 1**).

### EIVM data analysis

Manually analysis of digital video files was performed frame by frame at intervals of 0.04 s (25 frames) by ImageJ (National Institutes of Health). Venule diameters and cell velocities were measured for the hemodynamic parameters. Particularly, the velocities of 20 consecutive freely-flowing cells per vessel were manually measured for the determination of the fastest one. From the velocity of the fastest cell of each analyzed vessel (V_max_), we calculated the mean blood flow velocities (V_m_) according to equation: V_m_ = V_max_/(2 – ε 2) were ε is the ratio of lymphocyte diameter to the vessel diameter and the critical velocity (V_crit_), which was determined by using the following equation: V_crit_ = V_m_ × ϵ × (2 − ϵ). Wall shear stress (WSS) was calculated as wall shear rate (WSR) multiplied by an assumed blood viscosity of 0.025 Poise where WSR is determined by the formula: WSR = 8 × V_m_/vessel diameter. At least 100 consecutive cells/venule were examined to determine the cell fraction that was rolling or performed arrest within a given vessel. A cell was considered as rolling if traveled at a velocity lower than the V_crit_ whereas the arrest was considered when a cell remained stationary on the venule wall for ≥ 30 seconds (11, 21, 22).

### TPLSM imaging

Th1 or Th17 cells were labeled for 45 min at 37°C with 7-amino-4-chloromethylcoumarin (CMAC) (Molecular Probes). Then, 2 × 10^7^ Th1 or Th17 cells were intravenously injected into MOG_35-55_-immunized EAE recipient mice at the onset of the disease. After 48 h, when mice reached the EAE peak, 70 KDa fluorescein isothiocyanate dextran (FITC–dextran) (Sigma Chemical Co., St. Louis, MI, USA) was intravenously injected immediately before imaging of blood vessels. A mode-locked Ti : Sapphire Chameleon Ultra II laser (Coherent Inc.) was tuned to 750–800 nm and an Olympus XLUMPlanFI 20× objective (water immersed, numerical aperture, 0.95) was used for TPLSM imaging. To visualize infiltrating T cells in the dorsal spinal SAS, time-lapse sequences were performed at 70 μm stack height (2.5 μm z step) at 35-40 s intervals as previously described (17). Immediately after the first acquisition, 200 μl of artificial CSF containing 100 μg/ml of blocking anti-α4 integrins (clone PS/2, rat anti-mouse) or blocking anti-α4β7 integrin (clone DATK32, rat anti-mouse) were locally applied to the exposed spinal cord and incubated for 30 min before a second round of imaging, to allow adequate local meningeal diffusion (17, 23–26).

### TPLSM data analysis

Tracks longer than 3 min (≥ 12 time points) were manually tracked overtime from multidimensional rendering obtained by Imaris software (Bitplane) as previously described (17). Cell velocity and arrest index were computed. Track velocity was obtained from the mean instantaneous velocity calculated from all time intervals throughout a track. The arrest index represented the proportion of time during tracking in which a cell does not move (threshold ≤ 2 µm/min) and was calculated from cell tracks, so the reported value represents a percentage of cells in the entire population. The displacement of a cell moving with a constant velocity from an initial position, but randomly changing direction, was on average linearly proportional to the square root of the elapsed time (22).

### EAE induction and antibody treatment

C57BL/6J females (8–10 weeks old) were immunized subcutaneously in the flanks with 100 μg of MOG_35-55_ peptide in 200 μl emulsion containing phosphate-buffered saline (PBS) and complete Freund’s adjuvant (CFA; Difco Laboratories) in equal volumes, supplemented with 0.8 mg/mouse *Mycobacterium tuberculosis* strain H37Ra (Difco Laboratories). Mice were treated with 25 ng of pertussis toxin (Alexis Biochemicals) intravenously at the time of immunization and 48 h later. Clinical scores were recorded daily as previously described (27).

Anti-α4β7 treatment was administered intracisternally one day after the appearance of the first clinical signs and two days later with 50 μg of anti-α4β7 (clone DATK32, rat anti-mouse) or control antibody (clone Y13-259, rat anti-human/mouse/rat). Each mouse was anesthetized and the atlanto-occipital membrane was punctured with a Hamilton syringe fitted with a 27-gauge needle containing 10 μl of antibodies solution (1.4 mg/ml corresponding to 50 μg antibody in approximatively 35 µl of CSF) (28). We exclude potential interference with CNS leukocyte recruitment by antibody efflux from the CSF to periphery as discussed in our previous studies (17).

### Neuropathology

After 3 days from the first intrathecal antibody injection, EAE mice were euthanized for spinal cords explant and freezing. 20-μm sections were used for inflammatory infiltrates investigation by hematoxylin/eosin (29) and the area covered by infiltrating leukocytes was expressed as % of the total white matter area. Demyelination was evaluated by immunofluorescence staining with a purified anti-MBP antibody (clone D8X4Q, Cell Signaling), with DAPI as a nuclear co-stain. For the quantification of neuropathological findings, different lumbar and thoracic levels were evaluated on 4–6 cross-sections per mouse and results were expressed as a percentage of white matter area or total area, respectively (30).

For the quantification of infiltrating CD3^+^ cells, frozen sections of spinal cord were stained with an Alexa488-conjugated anti-CD3 antibody (clone 17A2, Biolegend) diluted 1:700, with DAPI as a nuclear co-stain. Results were represented as the area (µm^2^) of fluorescent CD3^+^ cells relative to the total spinal cord area. All images were captured using Axio Imager Z2 (Zeiss, Germany) and interactively quantified using Intellesis module of Zen 2.6 Lite after supervised training of the machine learning system.

### Statistics

Data from flow cytometry, EIVM and TPLM studies are expressed as means ± standard deviation (SD) or standard error of the mean (SEM). Statistical significance of data from flow cytometry and TPLM was calculated using the Mann–Whitney *U*-test, with a confidence interval of 95%. Outliers identification was calculated by Grubb’s test with a P value < 0.05 considered as statistically significant. Data from EIVM experiments were evaluated by ordinary one-way ANOVA followed by Dunnett’s multiple comparison. In the plots of displacement, we applied linear regression and compared the slopes.

## Results

### Hemodynamic factors are altered in the spinal cord pial vessels during EAE

**Figure 1:**
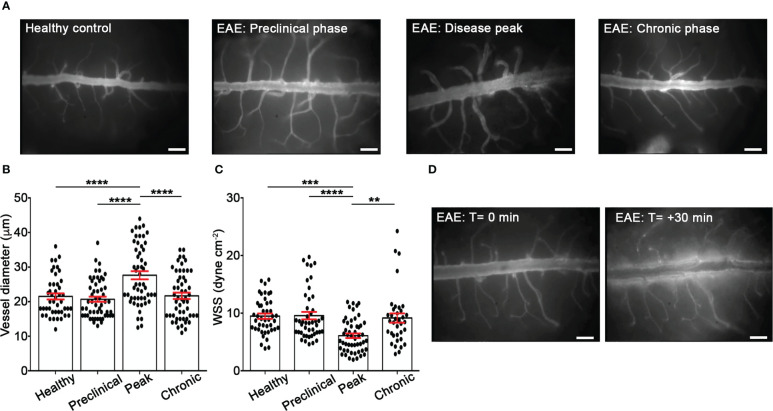
EIVM experiments show alterations of rheological factors in the spinal cord venules during EAE. C57BL/6 mice were injected with FITC dextran to visualize blood vessels. **(A)** Representative images of spinal cord pial venules (dorsal spinal vein fed by dorsal ascending venules) in healthy control animals and EAE mice at 9 (preclinical phase), 13-15 (disease peak considered 2 days after the appearance of the first clinical signs) and 22-25 (chronic phase) days post-immunization (dpi) (scale bar = 100 μm). **(B)** Diameters and **(C)** wall shear stress of pial vessels were measured in healthy controls and EAE mice at different time points of disease as described in material and methods. **(D)** Representative images of vascular leakage of FITC dextran at the disease peak. (**B**, **C**) Data are represented as mean ± SEM from a minimum of 30 to a maximum of 50 vessels from three independent experiments for each condition. One-way ANOVA followed by Tukey’s multiple comparison test were used for statistical analysis (*P = 0.0276; **P = 0.0055; ***P = 0.0003; ***P < 0.0001).

Rheological conditions have been shown to influence leukocyte and endothelium deformability, stiffness and activation and, consequently, leukocyte migration phenomena such as margination, rolling, integrin activation and diapedesis (31–33). To check the hemodynamics in EAE *versus* healthy mice, we performed laminectomy at the level of the lumbar spinal cord to visualize pial microcirculation. Our data clearly showed vasodilation and increased vessel diameter at disease peak compared with healthy animals, whereas no changes were observed at other disease time points (**Figures 1A, B**). In addition, we obtained results showing a reduction of wall shear stress (WSS) at disease peak compared to healthy mice and other disease conditions, further supporting an altered hemodynamic environment by acute/subacute inflammatory events during early clinical disease (**Figure 1C**). Furthermore, we observed a significant vascular leakage of the fluorescent tracker at disease peak, indicating profound inflammatory vascular changes potentially accompanied by leukocyte adhesion phenomena (**Figure 1D**).

### Th1 and Th17 cells have different adhesion capabilities in inflamed spinal cord pial vessels

Th1 and Th17 cells are potent inducers of autoimmunity and inflammation, and we next studied their adhesion capabilities in inflamed spinal cord leptomeningeal venules at different disease time points. By using flow cytometry analysis, we first studied the adhesive molecule expression of *in vitro* polarized MOG_35-55_-specific Th1 and Th17 cells in terms of percentage of positive cells and mean fluorescence intensity (MFI). The following adhesion molecules were investigated: α4 integrin chain, α4β7 and LFA-1 integrins, CD44, L-selectin and PSGL-1 mucin. Th17 cells showed a significantly lower expression of L-selectin (70.8% for Th1 *versus* 32.5% for Th17 cells, *P* = 0.0024) and higher levels of CD44 integrin (MFI 35.7 for Th1 cells *versus* 62.3 in Th17cells, *P* = 0.0452) suggesting a higher activation state compared to Th1 cells. Moreover, the percentage of Th17 cells expressing α4β7 integrin was higher compared to Th1 cells (54% for Th17 cells *versus* 31.4% for Th1 cells, *P* = 0.0196) (**Supplementary Figure 1**; **Supplementary Table I**), suggesting a potential role for this adhesion molecule in Th17 cell adhesion. LFA-1 was more expressed on Th1 cells compared to Th17 cells, further suggesting different adhesion potential and extravasation capacity of these two T cell types in the inflamed target tissue.

**Figure 2:**
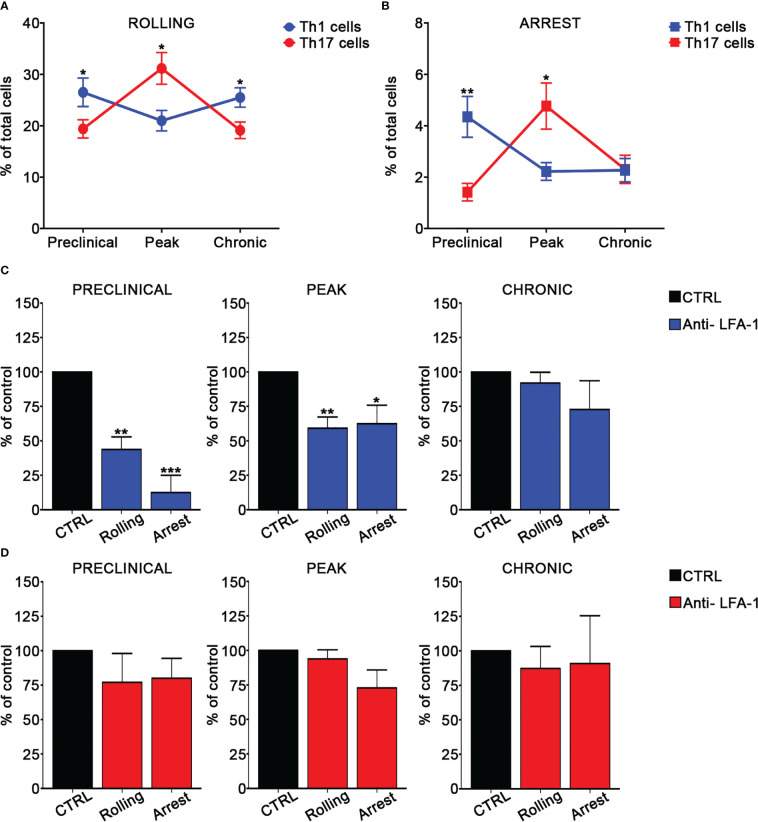
Th1 and Th17 rolling and arrest in spinal cord pial vessels during EAE. **(A, B)** *In vitro* differentiated Th1 and Th17 cells were fluorescently labeled and co-injected in immunized mice immediately before EIVM imaging at different time point of EAE. The percentage of rolling **(A)** and arrest **(B)** of Th1 and Th17 cells was evaluated as described in material and methods. Differences in terms of percentage of rolling and adhesion between Th1 and Th17 cells at different time points were determined by using Mann-Whitney *U* test (one tailed). Data are expressed as mean ± SEM from three independent experiments for each time point during EAE. (*P < 0.05; ** *P* = 0.0026). **(C, D)** *In vitro* differentiated Th1 or Th17 cells were fluorescently labeled, divided in two groups (untreated control cells and pretreated with the anti-LFA-1 antibody) and sequentially injected in immunized mice at different time points of EAE. EIVM imaging was performed immediately after every single cell injection at different time points of EAE. **(C)** Rolling and arrest of Th1 cells treated or not (CTRL) with anti-LFA-1 antibody at the preclinical phase, disease peak (2 days after the appearance of the first clinical signs) and chronic EAE. **(D)** Rolling and arrest of Th17 cells treated or not (CTRL) with anti-LFA-1 antibody at the preclinical phase, disease peak and chronic phase of EAE. One-way ANOVA followed by Dunnett’s multiple comparison test was applied to compare the frequency of rolling and adhesion events after antibody treatment with of untreated control cells (considered 100%). Data are represented as mean ± SEM from a minimum of 10 to a maximum of 19 venules from three independent experiments for each condition. (*P < 0.05, **P < 0.01; ***P < 0.001).

In order to investigate the adhesion of MOG_35-55_-specific Th1 and Th17 cells in the spinal SAS venules, we next performed EIVM experiments at different time points after EAE induction by simultaneous injection of Th1 and Th17 cells labelled with different fluorescent dyes. Particularly, we investigated T cell adhesion events in the pre-clinical phase at nine days post immunization (dpi), at disease peak (13–15 dpi), which was considered at two days after the onset of clinical signs, and during the chronic phase (22–25 dpi). Th1 cells showed a higher capacity to roll on pial vessel endothelium (26.5% *versus* 19.4%, *P* = 0.0172) and arrest (4.4% *versus* 1.4%, *P* = 0.0026) in leptomeningeal venules when compared to Th17 cells at the preclinical phase of disease. Conversely, at disease peak, Th17 cells were more adhesive when compared to Th1 cells, presenting a more efficient capacity to roll (26.1% *versus* 21.0% respectively, *P* = 0.0412) and arrest (3.8% *versus* 2.2% respectively, *P* = 0.0419). EIVM experiments performed during the chronic phase showed that Th1 and Th17 cells display a similar capacity to arrest in the spinal pial venules (2.3% *versus* 2.8%, *P* = 0.3705), although Th1 cells had a higher tendency to roll compared to Th17 cells (25.5% *versus* 20.0%, *P* = 0.0131) (**Figure 2A, B**; **Supplementary Table II**). Together, these data suggest a phase-specific adhesion capacity of Th1 and Th17 cells in disease development in EAE mice, prompting us to hypothesize a role for integrins in this phenomenon potentially relevant for the development of CNS inflammation.

### LFA-1 and α4β7 integrins have differential roles in Th1 and Th17 cell adhesion in leptomeningeal venules during EAE

We next evaluated the role of LFA-1 and α4 integrins in MOG_35-55_-specific Th1 and Th17 cell adhesion in spinal pial venules by performing EIVM experiments at different time points of EAE. T cell pre-treatment with an anti-LFA-1 blocking antibody led to a strong inhibition of both rolling and arrest of Th1 cells in the preclinical phase of EAE (57% blockade of rolling, *P* = 0.0030 and 87.5% inhibition of arrest, *P* = 0.0001). However, LFA-1 blockade had a less impact on adhesion interactions at disease peak and had no significant effect during the chronic phase, suggesting a prominent role for this integrin in Th1 cell migration during early EAE (**Figure 2C**; **Supplementary Table III**). Notably, LFA-1 blockade did not significantly affect the adhesive interactions between Th17 cells and vascular endothelium in the spinal SAS during all examined time points, demonstrating the existence of specific molecular mechanisms controlling Th1 *versus* Th17 cell adhesion in inflamed leptomeningeal vessels during EAE (**Figure 2D**; **Supplementary Table IV**). An isotype control antibody had no effect on the adhesion events of both Th1 and Th17 cells (data not shown).

**Figure 3:**
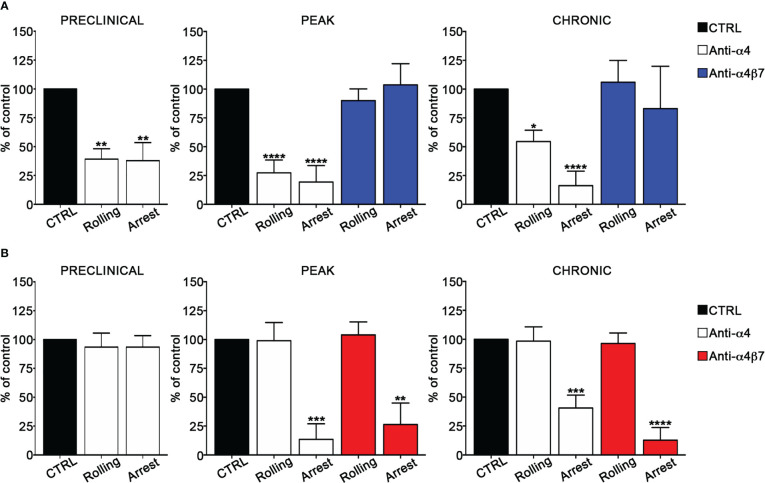
α4β7 blockade selectively reduces Th17 cells arrest in the spinal cord pial vessels during EAE. *In vitro* differentiated Th1 or Th17 cells were fluorescently labeled and sequentially injected in immunized mice in two tranches (untreated control cells and pretreated with the blocking antibody), EIVM imaging, conducted immediately after every single cell injection, was performed at the preclinical phase (9dpi), disease peak (13-15 dpi) and chronic phase (22-25 dpi) of EAE. **(A)** Rolling and arrest of Th1 cells before and after anti-α4 or anti-α4β7 antibodies treatment.**(B)** Rolling and arrest of Th17 cells before and after anti-α4 or anti-α4β7 treatment. Ordinary one-way ANOVA followed by Dunnett’s multiple comparison test was applied comparing the frequency of rolling and adhesion events after antibody treatment with control (considered 100%). Data are represented as mean ± SEM from a minimum of 12 to a maximum of 24 venules from three independent experiments for each condition. (*P < 0.05, **P < 0.01; ***P < 0.001, ****P < 0.0001).

We also determined the role of α4 integrins in EIVM experiments by using blocking anti-α4 or anti-α4β7 antibodies. In the preclinical phase of disease, α4 chain blockade in Th1 cells inhibited approximatively 60% of rolling and arrest events (*P* = 0.0027 and *P* = 0.0023 for rolling and arrest, respectively) (**Figure 3A**), whereas, in contrast, had no effect on Th17 cell adhesion (**Figure 3B**). Furthermore, α4β7 integrin blockade did not affect Th1 cell adhesiveness, suggesting VLA-4/α4β1 as the specific a4 integrin involved in Th1 cell rolling and adhesion in spinal pial vessels at the preclinical phase of disease (**Supplementary Figure 2**). At disease peak, α4 blockade led to a substantial reduction of rolling (73% inhibition, *P* < 0.0001) and arrest (81% inhibition, *P* < 0.0001) (**Figure 3A**) in Th1 cells. However, α4β7 blockade at the same time point of disease had no effect on Th1 adhesion, clearly indicating the predominant role of VLA4 integrin in Th1 cell intravascular adhesion during early clinical disease. Interestingly, α4 chain inhibition in Th17 cells massively reduced the arrest (87% inhibition, *P* = 0.0006), but had no effect on rolling, suggesting that different α4 integrins may control adhesion interactions in Th1 *versus* Th17 cells in inflamed leptomeningeal vessels (**Figure 3B**). Indeed, selective α4β7 blockade drastically inhibited the arrest (74% inhibition, *P* = 0.0037) without interfering with rolling, demonstrating a clear overlap with the results obtained with the anti-α4 chain blocking antibody and indicating a crucial role for α4β7 in Th17 cell adhesion in SAS vessels. Finally, we studied the adhesion interactions during the chronic phase of disease and observed that the pretreatment of Th1 cells with the anti-α4 chain antibody inhibited 46% of rolling (*P* = 0.0290) and almost completely abrogated the arrest (94% of inhibition, *P* < 0.0001). As also shown for the disease peak, α4β7 blockade had no effect on adhesion interactions, further supporting a pivotal role for VLA-4 in Th1 cell migration in the animal model of MS (**Figure 3A**; **Tables III**, **IV**). When we studied Th17 cell adhesion, we observed that the blockade of both α4 chain and α4β7 integrin dimer led to a drastic inhibition of arrest (60% inhibition, *P* = 0.0001 in the case of anti-α4 antibody and 88% inhibition, *P* < 0.0001 after α4β7 integrin blockade), further demonstrating the importance of α4β7 in Th17 cell stable adhesion (**Figure 3B**; **Tables III**, **IV**). The number of adhering controls Th17 cells during all EAE phases was generally low (from 1.1% of the total number of analyzed cells per vessel at the preclinical phase to 5.5% at the disease peak, (**Supplementary Table IV**) suggesting that Th17 cell arrest is tightly regulated. This observation, together with the strong inhibition exerted by α4β7 blockade, highlight the crucial role of α4β7 integrin in Th17 cell arrest in spinal leptomeningeal vessels. Collectively, these data also highlight a different usage of α4 integrins in T cell adhesion in inflamed leptomeningeal vessels, with VLA-4/α4β1 integrin being crucial for Th1 cells and α4β7 integrin having a pivotal role for Th17 cells.

### α4β7 integrin selectively controls Th17 cell motility in the spinal SAS during EAE

**Figure 4:**
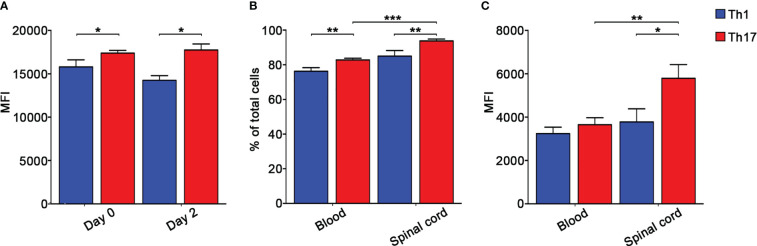
Endogenous and *in vitro* polarized Th17 cells express higher levels of α4β7 integrin compared to Th1 cells. **(A)** CMAC-labelled *in vitro* polarized Th1 or Th17 cells were intravenously injected into different EAE recipient mice at disease onset and harvested from the spinal cord 48 hours later. α4β7 integrin expression by Th1 (blue) and Th17 (red) cells was evaluated before and after cell transfer and shown as MFI. Data are expressed as mean ± SEM from 3-4 mice for each Th cell subset. Statistical significance was calculated using the Mann–Whitney test (*P < 0.05). Endogenous Th cell subpopulations were identified according to their chemokine receptors expression patterns. Particularly, CXCR3+ CCR4- CCR6- CD4+ T cells were classified as Th1 cells, whereas CXCR3- CCR4+ CCR6+ CD4+ T cells were classified as Th17 cells. **(B)** The percentages of α4β7 integrin-expressing endogenous circulating and spinal cord-infiltrating Th1 (blue) and Th17 (red) cells were evaluated by flow cytometry after surface staining. Data are expressed as mean ± SEM from 5-8 mice. Statistical significance was calculated using the Mann–Whitney test (**P < 0.01; *** *P* < 0.001). **(C)** MFI shows that endogenous spinal cord infiltrating Th17 cells display the highest level of α4β7 integrin. Data are expressed as mean ± SEM from 5-8 mice. Statistical significance was calculated using the Mann–Whitney test (*P < 0.05; ** *P* < 0.01).

To further understand the role of integrins in MOG_35-55_-specific Th1 and Th17 cell migration during EAE, we next determined the role of α4 integrins in the dynamics of extravasated Th1 and Th17 cells in the spinal SAS. Previous results obtained by our laboratory demonstrated that Th1 and Th17 cells massively infiltrate leptomeninges at disease peak during EAE showing significant differences in their motility behavior and suggesting distinct molecular mechanisms controlling their dynamics (17). In light of these data, we performed TPLSM experiments to evaluate the role of α4 integrins in the motility of Th1 and Th17 cells at disease peak. Particularly, we intravenously injected fluorescently labelled Th1 or Th17 cells into EAE mice at disease onset and, after 48 h when mice reached the disease peak. As previously shown for LFA-1 and α4 integrins (17), α4β7 expression was maintained on transferred exogeneous T cells after migration into the spinal cord, with Th17 cells having a higher expression of this integrin compared to Th1 cells (**Figure 4A**). To check if our *in vitro* differentiated T cells mimic the expression of α4β7 on endogenous cells, we evaluated α4β7 expression on endogenous Th1 and Th17 cells in the blood and spinal cord of EAE mice at disease peak. We identified CXCR3+ CCR4- CCR6- as Th1 cells and CXCR3- CCR4+ CCR6+ as Th17 cells (34). Our data confirmed that also endogenous Th17 cells have a higher α4β7 integrin expression compared to Th1 cells in terms of both percentage of expressing cells and MFI (**Figures 4B, C**).

**Figure 5:**
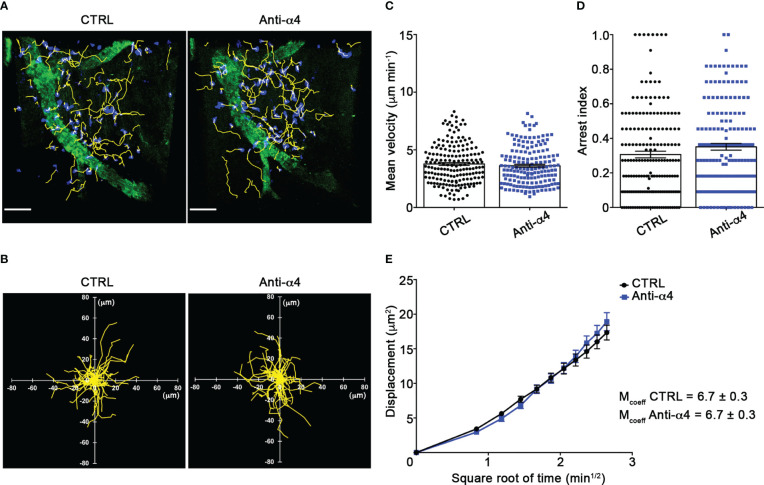
α4 integrins do not affect Th1 cells motility behavior in the spinal SAS at EAE peak. *In vitro* differentiated Th1 cells were fluorescently labeled and intravenously injected at disease onset. TPLSM imaging was performed after 48 h, when mice reached the disease peak. **(A)** Representative images of Th1 cells moving in the spinal SAS before (CTRL) and after 30 min of anti-α4 integrin treatment (anti-α4 antibody) (scale bar = 50 µm). **(B)** Normalized trajectories of 70 Th1 cells over 12 time points before and after anti- α4 antibody. **(C)** Mean velocity and **(D)** arrest index of Th1 cells before and after α4 integrins blockade. Statistical significance was calculated using the Mann–Whitney test. **(E)** Mean displacement of Th1 cells in not affected by anti-α4 blocking antibodies (After linear regression application, the differences between slopes resulted not quite significant; *P* = 0.0884). All data are expressed as mean ± SEM. A total of 185 cells were analyzed for CTRL and 171 after anti-α4 treatment from two independent experiments. Track lower than 12 time points were excluded from the analysis.

**Figure 6:**
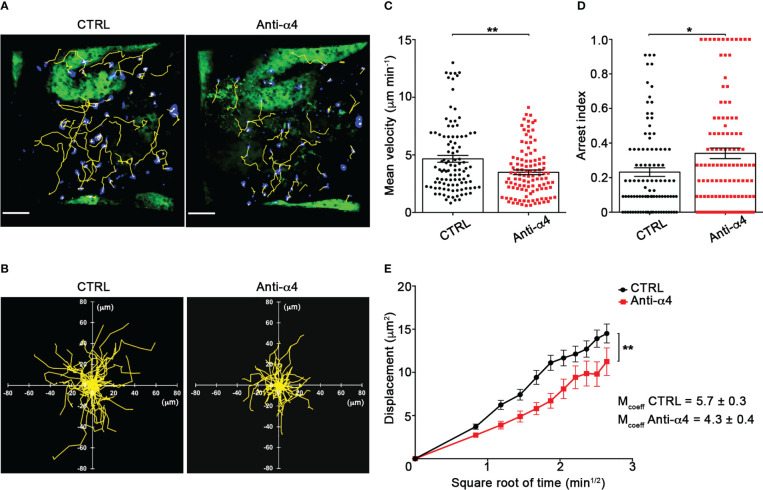
α4 integrins control Th17 cells motility in the spinal SAS at disease peak. *In vitro* differentiated Th17 cells were fluorescently labeled and intravenously injected at disease onset. TPLSM imaging was performed after 48 h, when mice reached the disease peak. **(A)** Representative images of Th17 cells before (CTRL) and after 30 min of anti-α4 integrin treatment (anti-α4 antibody) (scale bar = 50 μm). **(B)** Normalized trajectories of 70 Th17 cells over 12 time points before and after anti-α4 antibody. **(C)** Mean velocity (**P = 0.0095) and **(D)** arrest index (*P = 0.0149) of Th17 cells before and after α4 integrin blockade. Statistical significance was calculated using the Mann–Whitney test (*P < 0.05). **(E)** Mean displacement of Th1 cells is significantly reduced after anti-α4 blocking antibodies (After linear regression application, the differences between slopes resulted statistically significant; **P = 0.0072). All data are expressed as mean ± SEM. A total of 104 cells were analyzed for CTRL and 111 after anti-α4 treatment from two independent experiments. Track lower than 12 time points were excluded from the analysis.

**Figure 7:**
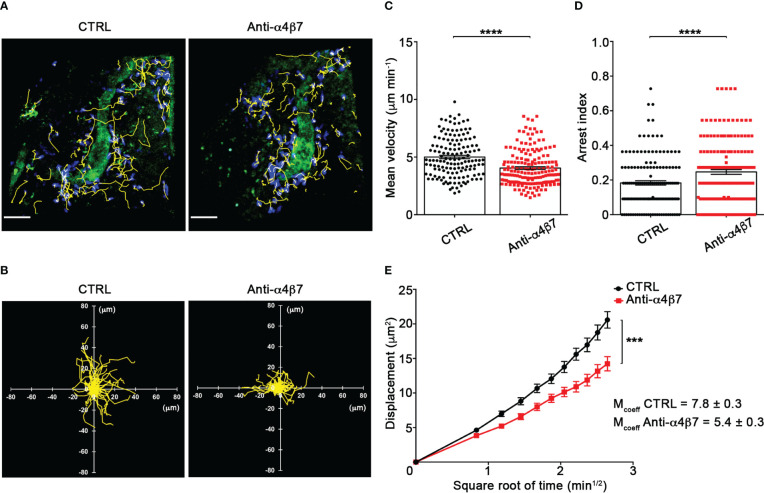
α4β7 integrin selectively controls Th17 cells dynamics in the spinal SAS at disease peak. Cell preparation and TPLSM imaging were perform as described for **Figure 5**. **(A)** Representative images of Th17 cells before (CTRL) and after 30 min of anti-α4β7 integrin treatment (anti-α4β7 antibody) (scale bar = 50 μm). **(B)** Normalized trajectories of 70 Th17 cells over 12 time points before and after anti-α4β7 antibody. **(C)** mean velocity and **(D)** arrest index of Th17 cells before and after integrin blockade. Statistical significance was calculated using the Mann–Whitney test (****P < 0.0001). **(E)** Mean displacement of Th17 cells is strongly decreased by anti-α4β7 blocking antibodies (After linear regression application, the differences between slopes resulted extremely significant; ***P < 0.0001). Data are expressed as mean ± SEM. A total of 146 cells were analyzed for CTRL and 176 after anti-α4 treatment from three independent experiments. Track lower than 12 time points were excluded from the analysis.

Our TPLSM experiments comparing cell motility before and after direct application of blocking antibodies on the exposed spinal cord, demonstrated no significant differences in the dynamics of Th1 cells before and after local α4 chain blockade, as shown by the measurements of mean velocity (3.8 *versus* 3.6 µm/min, *P* = 0.3622), arrest coefficient (0.31 *versus* 0.35, *P* = 0.0590) and displacement (curve slope: 6.*7 versus* 7.5, *P* = 0.0884) (**Figure 5**; **Supplementary Movie 2**), clearly indicating that α4 integrins are not involved in the locomotion of Th1 cells inside the spinal SAS of EAE mice. However, Th17 cells showed a significant motility reduction after α4 integrin blockade, as proved by the results on the Th17 cell speed (from 4.7 to 3.0 μm/min, *P* = 0.0095) (**Figure 6C**) and arrest index (0.23 *versus* 0.34, *P* = 0.0149) (**Figure 6D**). Moreover, Th17 cells showed a significant reduction in the curve slope (from 7.5 to 4.3, *P* = 0.0072), indicating that α4 integrin blockade interferes with their capacity to travel for long distances and cover large tissue volumes (**Figures 6A, B, E**). To unveil a potential role for α4β7 in Th17 cell motility in the spinal SAS, we next performed TPLSM experiments by directly applying a blocking anti-α4β7 antibody on the exposed spinal cord. Notably, our results clearly showed a strong reduction in the movement velocity of Th17 cells after α4β7 blockade (5.0 *versus* 4.0 μm/min, *P* < 0.0001), an increment of the arrest index (from 0.28 to 0.43, *P* < 0.0001) and a decrease of the slope of displacement curve (from 7.8 to 5.4, *P* < 0.0001), strongly indicating a major role for α4β7 integrin in the locomotion of Th17 cells inside the spinal SAS during EAE (**Figure 7**; **Supplementary Movie 3**). However, in the present study we observed some differences in the velocity of Th cells comparing to our previous data (17), probably due to variability in meningeal inflammation between mice and the fact that in this work we did not co-inject Th1 and Th17 cells in the same animals.

### Intrathecal α4β7 blockade inhibits the development of EAE

**Figure 8:**
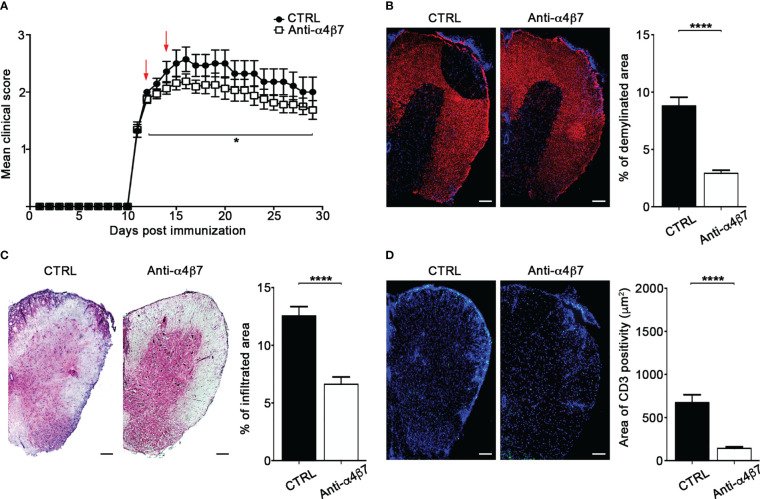
Intrathecal treatment with an anti-α4β7 blocking antibody ameliorates EAE. EAE mice were intrathecally injected with 10 μl PBS containing 50 μg of isotype control antibody (CTRL) or anti-α4β7 blocking antibody the day after disease onset and 2 days later. Some mice were euthanized 3 days after the first antibody injection for spinal cords neuropathology assessment. **(A)** EAE progression was measured by daily scores for the severity of clinical disease symptoms. Red arrows indicate intrathecal antibody administration. Data represent the mean ± SEM of 29 mice per condition from three independent experiments (*P < 0.05). Representative spinal cord sections and relative quantitative analysis of **(B)** demyelination, **(C)** inflammatory infiltrates and **(D)** CD3+ T cells, from mice treated with control (CTRL) or anti-α4β7 antibodies. 4–6 cross sections of spinal cord of 3 mice were analyzed. Error bars indicate SEM (****P < 0.0001). Scale bar = 100μm.

We have previously shown that interfering with autoreactive T cell motility hampers detrimental T cell activities and, consequently, attenuates EAE development (17). To determine the therapeutical effect of α4β7 integrin blockade on extravasated Th17 cell motility inside the spinal SAS, we intrathecally injected two doses of an anti-α4β7 antibody in EAE mice after the clinical onset (one day after the appearance of the first clinical signs and two days later). These doses were higher than the saturating dose of anti-α4β7 mAb found in our previous flow cytometry studies (data not shown). α4β7 blockade led to a statistically significant reduction in disease severity compared to a control antibody, and the effect lasted at least two weeks after treatment termination (*P* = 0.0461) (**Figure 8A**). Disease amelioration was associated with a marked reduction in the demyelination (*P* < 0.0001) and inflammatory cell infiltration (*P* < 0.0001) in the anti-α4β7 treated animals compared to isotype control treated mice (**Figures 8B, C**). Also, we found a significant reduction in the CD3^+^ cell compartment (*P* < 0.0001), suggesting that intrathecal α4β7 blockade also reduces T cell invasion of the CNS, attenuating the neuroinflammatory environment (**Figure 8D**).

## Discussion

MS and its experimental models are complex and heterogeneous chronic inflammatory disorders of the CNS with several immune cell populations involved in disease pathogenesis, including Th1 and Th17 cells (18, 35, 36). Indeed, previous studies suggested that disease heterogeneity in terms of different clinical course, CNS lesion distribution in the brain *versus* spinal cord and response to immunomodulatory therapies, may depend on whether the predominant immune response is mediated by Th1 or Th17 cells (35, 37–45).

Both human and murine Th1 and Th17 cells express different patterns of adhesion molecules and chemokine receptors that can influence the preferential accumulation of these cells in the brain or spinal cord (35). VLA-4 is considered the major integrin controlling the accumulation of Th1 cells in the CNS (46), while LFA-1 was shown to control Th17 cell recruitment into the brain in the absence of functional α4 integrins (47). Thus, the relative levels of integrin expression on Th1 and Th17 cells may support different patterns of migration and inflammation, leading not only to the preferential localization of these cells in the spinal cord *versus* brain, but also indirectly impacting the subsequent recruitment of other immune cell types contributing the inflammatory responses. Our *in vitro* polarized MOG_35-55_-specific Th17 cells had higher expression of CD44 and lower L-selectin expression, supporting an increased ability to invade non-lymphoid inflamed tissues (48, 49). Notably, the percentage of Th17 cells expressing α4β7 integrin was significantly higher compared to Th1 cells, and these data are supported by previous studies showing that α4β7^hi^ CD4+ T cells harbor most Th17 cells during viral infection (50). We did not find differences in the expression of α4 integrin chain between Th1 and Th17, as also supported by other studies (51). However, data obtained by Rothhammer et al. found a higher α4 integrin expression on *in vitro* differentiated Th1 cells compared to Th17 cells (47). This may be explained by a different *in vitro* protocol, in which Th cells were differentiated by a single polyclonal stimulation without subsequent cell amplification, whereas in our manuscript, as well as in other studies, naïve CD4+ T cells were primed with MOG_35-55_ twice followed by cell amplification with cytokines (47, 51). In our experimental setting, *in vitro* differentiated Th17 cells showed higher expression of α4β7 integrin compared to Th1 cells, and this difference was also found on endogenous Th cells migrated into the spinal cord, suggesting that our experimental system could mimic the motility capability of Th1 and Th17 cells during EAE, as also previously demonstrated by our group (17). The higher expression of α4β7 on exogenous transferred Th17 cells may be explained by the stronger activation induced by *in vitro* polarization, making them less responsive to the local inflammatory milieu, as indicated by their unperturbed integrin profile after 48 hours of *in vivo* migration. In addition, the lower expression of α4β7 on endogenous Th17 cells could be due to a more heterogenous state of activation of these cells during EAE. However, α4β7 expression on migrated endogenous Th17 cells is drastically enhanced by the spinal environment.

Contradictory data have been published on the sequence of Th1 and Th17 cell entry into the CNS during EAE development. Previous reports suggested that Th1 cells entry into the CNS is required to facilitate subsequent wave of Th17 cell migration (52), whereas other studies found that Th17 cells are the earliest subpopulation infiltrating the CNS in the pre-clinical phase, with Th1 cells accumulating later at disease onset (53, 54). Most likely, the collection of these data in EAE mice was influenced by the high *in vivo* plasticity shown by Th1 and Th17 cells (55–57). To overcome the limitation of a potential *in vivo* phenotype change, we used *in vitro* differentiated MOG_35-55_-specific Th1 and Th17 cells and studied their adhesion in short term assays in EIVM experiments immediately after intravenous injection. Also, MOG_35-55_-specific Th1 and Th17 cells were used in TPLSM studies within 2 days after injection, when these cells maintain their initial phenotype as previously described (17).

Whereas T cell accumulation was mainly investigated in the brain and spinal cord of EAE mice, growing evidence highlight a key role for the meningeal space in driving parenchymal immune cell invasion and inflammation in EAE and MS (10, 58–60). However, the molecular mechanisms controlling rapid adhesion events such as tethering, rolling and arrest in spinal cord leptomeninges as well as the migration behavior in the meningeal space during EAE are not well elucidated. In this manuscript we studied the ability of MOG_35-55_-specific Th1 and Th17 cells to interact with endothelium in spinal cord pial venules at different phases of EAE and our data found clear differences between these two cell populations, suggesting distinct molecular mechanisms regulating their extravasation into the meninges. Indeed, Th1 cells showed higher rolling and arrest capacity than Th17 cells during the preclinical phase of EAE, pointing to a prominent role for Th1 cells in leptomeningeal inflammation during early EAE. IFN-γ produced by Th1 cells may induce expression of adhesion molecules and tight junctions alterations, facilitating the migration of subsequent inflammatory cells as demonstrated by other studies (61–63). At disease peak, Th17 cells displayed a significantly higher capacity to perform arrest, suggesting a pathological role for Th17 cells necessary for clinical disease progression. Indeed, previous studies have shown that IL-17A induces blood-brain barrier dysfunction, favoring ensuing transmigration of other lymphocyte subpopulations, including Th1 cells, in the CNS (64–66). Vascular leakage observed in our study coincides with higher Th17 adhesion, further pointing to Th17 cells as key players in the progression of clinical disease. Some differences in rolling between Th1 and Th17 cells were observed during chronic EAE, but the arrest capacity was similar, suggesting that both cell types may contribute to chronic leptomeningeal inflammation.

Our data show that α4 integrins are key adhesion molecules mediating both rolling and arrest of Th1 cells in leptomeningeal vessels during all EAE phases (pre-clinical phase, disease peak and chronic phase), confirming the predominant role of α4 integrins in Th1 cell adhesion. Interestingly, blockade of α4 integrins in Th17 cells only reduced the arrest without interfering with rolling, suggesting these molecules may be differently distributed on the plasma membrane of Th17 cells compared to Th1 cells (67). Moreover, these data also hinted that distinct α4 integrins may be involved in Th17 *versus* Th1 cell adhesion in leptomeningeal vessels. Indeed, our results clearly showed that a blocking antibody against α4β7 integrin strongly inhibited the adhesion of Th17 cells at disease peak and during the chronic phase, but had no effect on Th1 cells, unveiling a selective role for α4β7 integrin in Th17 cell migration in leptomeningeal vessels. In support of our data, a selective role for α4β7 integrin in Th17 trafficking has also been shown in a murine model of autoimmune ocular inflammation (51). In addition, previous studies reported that in *Rag1 ^-/-^* mice in which EAE was induced by a transfer of *in vitro* differentiated MOG_53-55_-specific Th17 cells, systemic blockade of α4 integrins inhibited Th17 migration into the spinal cord parenchyma, but not the brain inducing signs of atypical EAE with ataxia and hemiparesis. These data were also confirmed in EAE mice by transferring α4 deficient Th17 cells. Interestingly in the same experimental setting, the interference with α4 integrin chain completely abrogated transferred Th1 cell accumulation in both spinal cord and brain reducing EAE manifestations and further supporting a role for α4β7 integrin in Th17 cell migration during MOG_35-55_-induced EAE (47). However, α4 integrins had no role in Th17 adhesion in leptomeningeal vessel during the preclinical phase of disease, suggesting that other molecules are responsible for the entry of Th17 in the spinal SAS during this disease phase. Interestingly, the adhesion interactions of Th17 cells were not affected by LFA-1 blockade during all disease phases, whereas Th1 cell adhesion was significantly reduced, further demonstrating relevant differences in the migration mechanisms between Th1 and Th17 subsets. Indeed, previous studies suggested that LFA-1 may control Th17 migration into the brain, but not into the spinal cord, highlighting the complexity of Th cell recruitment process during EAE (47).

MAdCAM-1 is considered the high affinity ligand for α4β7 integrin (68). It was shown to be expressed on inflamed CNS vessels in a chronic relapsing form of EAE, but its role in T cell migration during EAE is not well understood (69–71). Whereas in a model of relapsing–remitting EAE in SJL mice, blockade of β7 integrins had no effect on disease (72), inhibition of MAdCAM-1 or β7 integrins significantly interfered with disease development in a chronic EAE model in C57BL/6 mice, supporting our data obtained in the same experimental model (70, 73). Although with lower affinity than VLA-4, α4β7 integrin can bind VCAM-1 (74), and we hypothesize that VCAM-1, which has a high endothelial expression in the CNS during MS and EAE, may represent an alternative endothelial counter-ligand in leptomeningeal vessels for α4β7 integrin expressed on Th17 cells (75).Once migrated through the pial vessel wall, encephalitogenic T cells need to be reactivated by leptomeningeal APCs for acquiring the ability to infiltrate the CNS parenchyma (10, 76). In this context, T cell locomotion inside the SAS could be therefore of fundamental importance for antigen scanning and contacts with resident cells. However, our understanding of activated T cell dynamics after extravasation into the CNS and meninges is still unclear. By using a TPLSM approach, we have previously shown two distinct LFA-1-dependent migratory patterns used by Th1 and Th17 cells, suggesting that different molecular mechanisms control intrameningeal locomotion during EAE (17). Also, our data demonstrated that interfering with T cell motility inside the spinal SAS reduces their pathogenic potential and mitigates EAE (17). In a murine model of autoimmune ocular inflammation, Cox and colleagues reported that *in vivo* reactivation leads to higher expression of α4β7 and CCR6 in transferred Th17, but not Th1, cells, further emphasizing the importance of migration mechanisms in CD4+ cell pathogenicity (51). In our TPLSM experiments, Th1 motility behavior in the leptomeninges was independent of α4 integrins, whereas α4 integrin chain blockade led to a drastic reduction of Th17 cell locomotion in terms of mean velocity, arrest index and displacement in the spinal SAS at EAE peak. α4β7 expression was higher on Th17 cells in our experimental setting, and, in line with this finding, blockade of α4β7 integrin significantly affected Th17 trafficking, further suggesting a critical role for this adhesion molecule in Th17 migration and leptomeningeal inflammation. It is known that in the absence of shear forces, such as those exerted during leukocyte extravasation, the induction of the high affinity state of integrins is not sufficient to support adhesive mechanisms during intratissue cell motility (77). Interestingly, it was previously demonstrated that, even in the absence of adhesive interactions, integrins are necessary for T cells locomotion mediating substrate friction between the actin cortex, controlled by chemokine receptors, and tissue environment (78), suggesting that this scenario may also verify during spinal SAS in EAE mice.

Previous studies have shown that α4 integrins are required for the maintenance of T cell “residency” in the CNS (79), suggesting this may be also the case for sustained permanence and pathological effects of Th17 cells in the meningeal space during EAE.

To further understand the relevance of Th17 α4β7 integrin in meningeal inflammation and EAE development, we treated mice immunized with MOG_35-55_ peptide (actively-induced EAE) by intrathecal administration of a blocking anti-α4β7 integrin antibody. Notably, our results show that two doses of anti-α4β7 antibody injected after disease onset were sufficient to significantly reduce disease severity and neuropathology, clearly substantiating the relevance of α4β7 integrin in T cell locomotion and leptomeningeal inflammation. As expected, α4β7 blockade also led to a reduction in CD3^+^ cell accumulation in the spinal cord without impacting T cells survival or activation, suggesting that therapeutic α4β7 inhibition reduces T cell invasion. This underlines once more the importance of T cell motility inside the leptomeningeal structures in disease development. Moreover, even if we cannot exclude that *in vitro* differentiated Th cells rely on α4β7 integrin more than endogenous ones, our intrathecal treatment approach, that resulted in a significant mitigation of EAE, further suggests our experimental approach as a reliable model of investigation. Intriguingly, the beneficial effect of anti-α4β7 blockade on disease lasted for a long time, highlighting the pivotal role of Th17 cells in the maintenance of inflammatory responses during chronic disease progression.

In conclusion, our results show that α4β7 integrin selectively controls Th17 cell adhesion in leptomeningeal vessels and trafficking inside the spinal cord SAS, potentially influencing subsequent parenchymal inflammation and contributing to vascular dysfunction and lesion distribution (80). Understanding the molecular mechanisms responsible for the specific contribution of Th1 and Th17 cells to the pathogenesis of autoimmune inflammatory disease of the CNS may help to identify new therapeutic strategies for these disorders.

Both EAE and neuromyelitis optica spectrum disorders (NMOSD) have been correlated to a preferential Th17 cells activity in optic nerves and spinal cord (81–83). However, although in both disorders Th17 migration into the spinal cord is considered a crucial pathogenic mechanism, natalizumab treatment in NMOSD patients proved ineffective (84), suggesting a more selective intervention on Th17 cells is needed. Also, the severe disease rebound showed by some RR-MS patients after long-term natalizubam treatment, is associated to a pathogenic profile in Th17 cells, including altered migratory features, further indicating that therapies aimed at interfering with Th17 cell function may be beneficial in MS (85). Interestingly, *in vivo* systemic blockade of β7 integrin subunit at disease peak during a transfer EAE model, induced a gradual disease remission. Moreover, when coinjected with anti-α4 antibody, this antibody potentiated the therapeutic effect of the anti-α4 treatment (73). Together, these data suggest that inhibition of α4β7 integrin may have some beneficial effects over the anti-α4 therapy, especially in patients with high numbers of circulating Th17 cells and therefore, at elevated risk of disease rebound post natalizumab (86). Thus, in the context of existing literature, our results suggest that anti-α4β7 antibodies, already in clinical use for subjects with inflammatory bowel diseases, may also be beneficial in patients with MS (87, 88).

## Data availability statement

The original contributions presented in the study are included in the article/**Supplementary Material**. Further inquiries can be directed to the corresponding authors.

## Ethics statement

The animal study was reviewed and approved by Institutional Animal Care Committee (OPBA) of the University of Verona.

## Author contributions

BR, SD, GA, AB, NL, VD, EP, EZ, and CZ, performed the research. BR analyzed the data. BR and GC designed the study and wrote the paper. All authors contributed to the article and approved the submitted version.

## Supplementary material

Supplementary Figure 1 Characterization of *in vitro* polarized MOG35-55-specific Th1 and Th17 cells. Th cell subpopulations were studied by flow cytometry to evaluate their cytokine production or adhesion molecules profile. (**A**) Representative gating strategy used to assess Th1 and Th17 cells for their specific patterns of cytokine production and trafficking receptors. (**B**) The percentages of IFN-γ+, IL-17+ and IFN-γ+ IL-17+ Th1 (left panel) and Th17 (right panel) cells were evaluated by intracellular flow cytometry staining, as described in materials and methods. Data are expressed as mean ± SEM from 12 independent experiments. (**C**) The percentages of Th1 (blue) and Th17 (red) cells expressing α4 integrin, α4β7 integrin, LFA-1 integrin, CD44, CD62L and PSGL-1 were assessed by flow cytometry after surface staining. Data are expressed as mean ± SD from 12 independent productions of each Th cell subset. Statistics were calculated using two tailed Mann–Whitney test (**P* = 0.0196; ****P* = 0.0024). (**D**) Representative histograms plot and quantification of the MFI of α4 integrin (upper left panel), α4β7 integrin (upper central panel), LFA-1 integrin (upper right panel), CD44 (lower left panel), CD62L (lower central panel) and PSGL-1 (lower right panel) displayed by Th1 (blue) and Th17 cells (red). Data are expressed as mean ± SD from 12 independent experiments of each Th cell subset. Statistics were calculated using two tailed Mann–Whitney test (**P* < 0.05).

Supplementary Figure 2 α4β7 integrin blockade does not affect Th1 cell rolling and arrest in the spinal cord pial vessels during preclinical EAE. *In vitro* differentiated Th1 cells were fluorescently labeled and intravenously injected into EAE recipient mice during the preclinical phase (9 dpi) of disease. EIVM imaging was conducted immediately after cell transfer. Rolling and arrest of Th1 cells were evaluated before and after anti-α4β7 antibody treatment. One-way ANOVA followed by Dunnett’s multiple comparison test were applied to compare the frequency of rolling and adhesion events after antibody treatment with control (considered 100%). Data are represented as mean ± SEM from a minimum of 12 to a maximum of 24 venules from three independent experiments.

Supplementary Table I Expression of adhesion molecules on Th1 and Th17 cells. Results are expressed as mean ± SD of 24 independent *in vitro* productions (12 for Th1 cells and 12 for Th17 cells). MFI represents mean fluorescence intensity. Statistics were calculated using two tailed Mann–Whitney test.

Supplementary Table II Diameter, hemodynamics, and rolling velocities of Th1 and Th17cells in the SC at the different phases of EAE. The mean blood flow velocity (Vm), wall shear stress (WSS), and the percentage of rolling and arrested cells were calculated as described in Materials and Methods. At least 100 consecutive cells/venule were examined. The velocity of rolling cells was measured by digital frame-by-frame analysis of videotapes. Vroll are presented as median. Data are arithmetic mean ± SD for hemodynamic parameters and mean ± SEM for the percentages of rolling and arrest.

Supplementary Table III Diameter, hemodynamics, and rolling velocities of Th1 cells treated with blocking antibodies. Vm, WSS, and the percentage of rolling and arrested cells were calculated as described in Materials and Methods. At least 100 consecutive cells/venule were examined. The velocity of rolling cells was measured by digital frame-by-frame analysis of videotapes. Rolling velocity (Vroll) are presented as median. Data are arithmetic mean ± SD for hemodynamic parameters and mean ± SEM for the percentages of rolling and arrest.

Supplementary Table IV Diameter, hemodynamics, and rolling velocities of Th17 cells treated with blocking antibodies. Vm, WSS, and the percentage of rolling and arrested cells were calculated as described in Materials and Methods. At least 100 consecutive cells/venule were examined. The velocity of rolling cells was measured by digital frame-by-frame analysis of videotapes. Rolling velocity (Vroll) are presented as median. Data are arithmetic mean ± SD for hemodynamic parameters and mean ± SEM for the percentages of rolling and arrest.

Supplementary Movie 1 Representative movie obtained by EIVM of Th1 rolling (white edge) or firmly adhering (white asterisk) on spinal pial vessels in a MOG_35-55_-immunized recipient mouse at disease peak. Th1 cells were intravenously injected immediately before the imaging. FITC-dextran was injected for vessel visualization and *in vitro* CMFDA-labeled Th1 cells were intravenously injected immediately before the imaging. Cells were manually analyzed frame by frame to determine the fraction of cells that were rolling or under firm adhesion on the total number of cells that entered that vessel. The fastest cell from 20 consecutive freely-flowing in each vessel was used to calculate hemodynamic parameters.

Supplementary Movie 2 Th1 cell dynamics in the spinal leptomeninges is not affected by anti-α4 integrin local blockade. Representative videos of antigen specific Th1 cells tracking inside spinal cord SAS, before (left) and after (right) anti-α4 integrins treatment in MOG_35−55_-immunized mice at the peak of EAE. Th1 cell motility is not affected after local blockade, suggesting that locomotion of Th1 cells inside the spinal SAS during the disease peak is α4-integrins independent. Vessels are visualized in green. Velocity barr = 0-0.2 µm/s.

Supplementary Movie 3 Anti-α4 integrin local blockade affected Th17 cell dynamics in the spinal leptomeninges. Representative videos of antigen specific Th17 cells tracking inside spinal cord SAS, before (left) and after (right) anti-α4 integrins treatment in MOG_35−55_-immunized mice at the peak of EAE. Th17 cell motility decreased after local blockade inside the spinal SAS during EAE peak, suggesting that Th17 cells locomotion is under α4 integrins handle. Vessels are visualized in green. Velocity barr = 0-0.2 µm/s.

Supplementary Movie 4 Local α4β7 integrin blockade of MOG_35-55_-specific Th17 cells significantly reduced their dynamics in the spinal cord SAS. Representative videos of antigen specific Th17 cells tracking inside spinal cord SAS, before (left) and after (right) anti-α4β7 integrin treatment in MOG_35−55_-immunized mice at the peak of EAE. Th17 cell motility is drastically reduced after local blockade, strongly indicating a critical role for α4β7 integrin in the locomotion of Th17 cells inside the spinal SAS during the disease peak. Vessels are visualized in green. Velocity barr = 0-0.2 µm/s.
