# Supplementary figures and images for: Alpha4 beta7 integrin controls Th17 cell trafficking in the spinal cord leptomeninges during experimental autoimmune encephalomyelitis
Source: Front Immunol. 2023 Apr 18;14:1071553. doi: 10.3389/fimmu.2023.1071553 (PMC10151683; doi:10.3389/fimmu.2023.1071553)

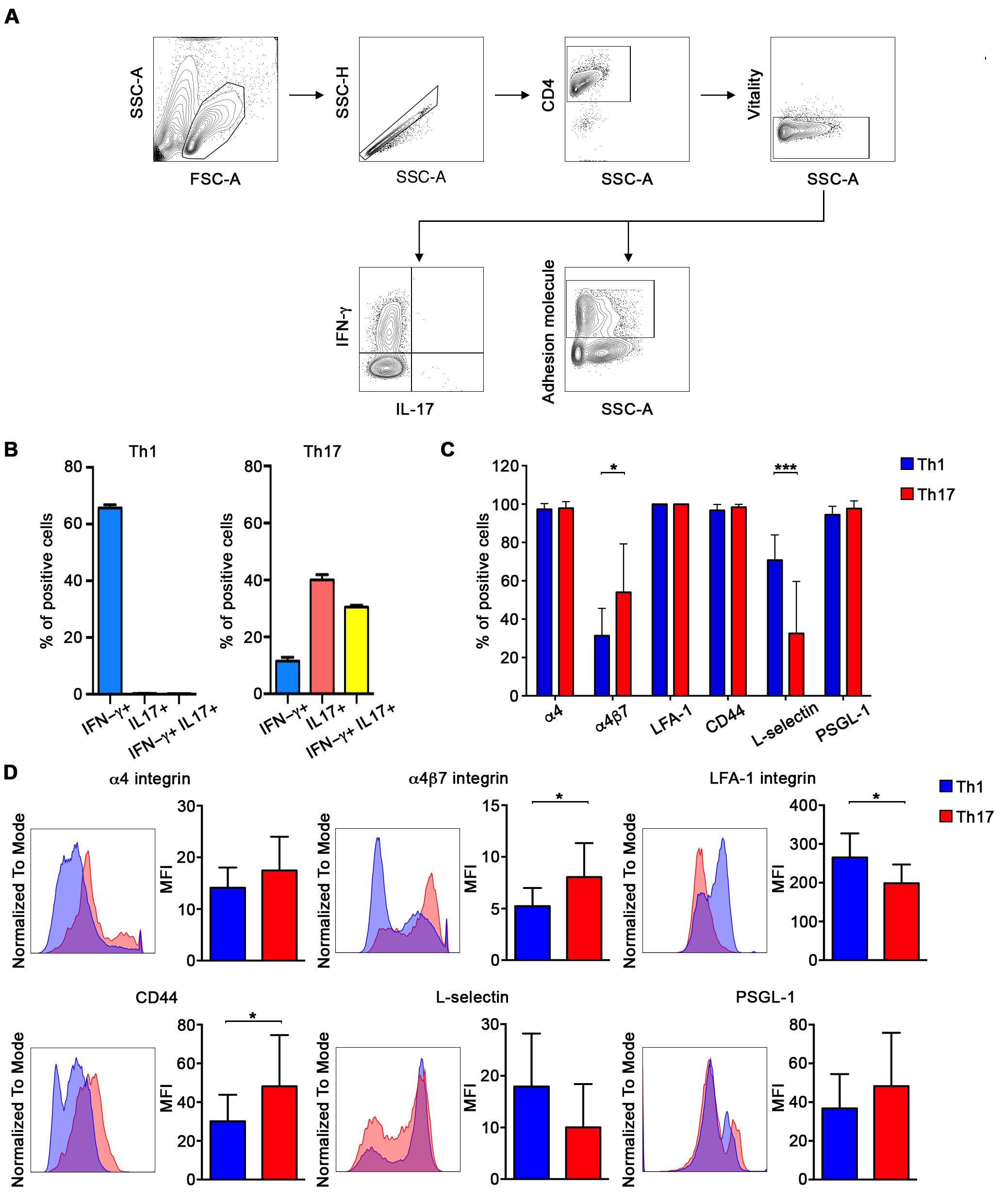

Supplement: Supplementary Figure 1 — Characterization of in vitro polarized MOG35-55-specific Th1 and Th17 cells. Th cell subpopulations were studied by flow cytometry to evaluate their cytokine production or adhesion molecules profile. (A) Representative gating strategy used to assess Th1 and Th17 cells for their specific patterns of cytokine production and trafficking receptors. (B) The percentages of IFN-γ+, IL-17+ and IFN-γ+ IL-17+ Th1 (left panel) and Th17 (right panel) cells were evaluated by intracellular flow cytometry staining, as described in materials and methods. Data are expressed as mean ± SEM from 12 independent experiments. (C) The percentages of Th1 (blue) and Th17 (red) cells expressing α4 integrin, α4β7 integrin, LFA-1 integrin, CD44, CD62L and PSGL-1 were assessed by flow cytometry after surface staining. Data are expressed as mean ± SD from 12 independent productions of each Th cell subset. Statistics were calculated using two tailed Mann–Whitney test (*P = 0.0196; ***P = 0.0024). (D) Representative histograms plot and quantification of the MFI of α4 integrin (upper left panel), α4β7 integrin (upper central panel), LFA-1 integrin (upper right panel), CD44 (lower left panel), CD62L (lower central panel) and PSGL-1 (lower right panel) displayed by Th1 (blue) and Th17 cells (red). Data are expressed as mean ± SD from 12 independent experiments of each Th cell subset. Statistics were calculated using two tailed Mann–Whitney test (*P < 0.05). [file Image_1.tif]

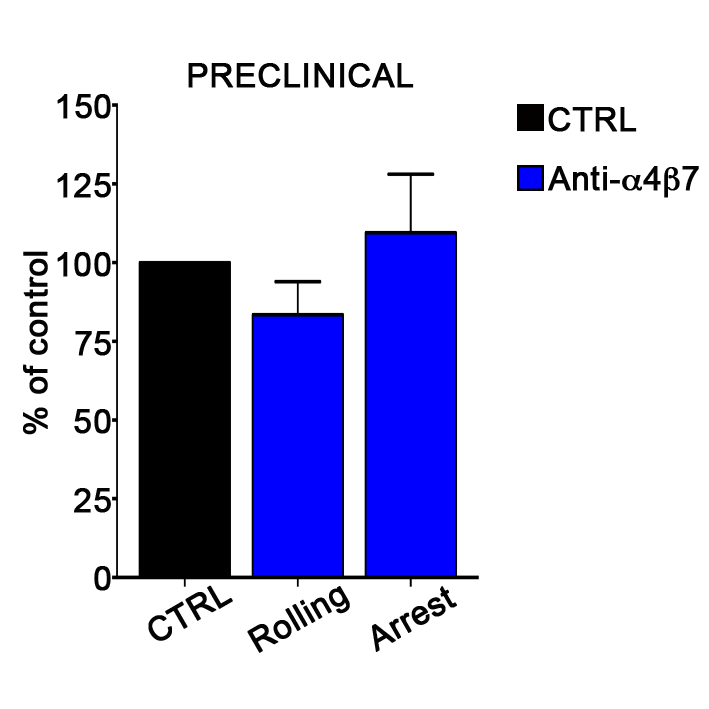

Supplement: Supplementary Figure 2 — α4β7 integrin blockade does not affect Th1 cell rolling and arrest in the spinal cord pial vessels during preclinical EAE. In vitro differentiated Th1 cells were fluorescently labeled and intravenously injected into EAE recipient mice during the preclinical phase (9 dpi) of disease. EIVM imaging was conducted immediately after cell transfer. Rolling and arrest of Th1 cells were evaluated before and after anti-α4β7 antibody treatment. One-way ANOVA followed by Dunnett’s multiple comparison test were applied to compare the frequency of rolling and adhesion events after antibody treatment with control (considered 100%). Data are represented as mean ± SEM from a minimum of 12 to a maximum of 24 venules from three independent experiments. [file Image_2.tif]
