# Supplementary material for: Alpha4 beta7 integrin controls Th17 cell trafficking in the spinal cord leptomeninges during experimental autoimmune encephalomyelitis
Source: Front Immunol. 2023 Apr 18;14:1071553. doi: 10.3389/fimmu.2023.1071553 (PMC10151683; doi:10.3389/fimmu.2023.1071553)
Supplement: Supplementary Table I — Expression of adhesion molecules on Th1 and Th17 cells. Results are expressed as mean ± SD of 24 independent in vitro productions (12 for Th1 cells and 12 for Th17 cells). MFI represents mean fluorescence intensity. Statistics were calculated using two tailed Mann–Whitney test. [file Table_1.pdf]

Supplementary Table I.

	Th1	Th17	
	**% of positive cells [MFI]**	**% of positive cells [MFI]**	***P* value**
**α4**	97.3 ± 3.0<br>[14.1 ± 3.9]	97.8 ± 3.4<br>[17.4 ± 6.5]	n.s.<br>[n.s.]
**α4β7**	31.4 ±14.2<br>[5.2 ±1.7]	54.0 ± 25.2<br>[8.0 ± 3.3]	***P = 0.0196**<br>[***P = 0.0309**]
**LFA-1**	99.9 ±0.1<br>[265.1 ±62.1]	99.8 ± 0.2<br>[198.6 ± 48.5]	n.s.<br>[***P = 0.0159**]
**CD44**	96.7 ± 3.1<br>[35.7 ±17.6]	98.3 ± 1.5<br>[62.3 ± 48.4]	n.s.<br>[***P = 0.0452**]
**L-selectin**	70.8 ± 13.1<br>[40.4 ± 15.0]	32.5 ± 27.1<br>[30.9 ± 16.0]	*****P = 0.0024**<br>[n.s.]
**PSGL-1**	94.5 ± 4.2<br>[38.4 ± 16.1]	97.7 ± 3.9<br>[67.2 ± 25.4]	n.s.<br>[n.s.]
