# Supplementary material for: Alpha4 beta7 integrin controls Th17 cell trafficking in the spinal cord leptomeninges during experimental autoimmune encephalomyelitis
Source: Front Immunol. 2023 Apr 18;14:1071553. doi: 10.3389/fimmu.2023.1071553 (PMC10151683; doi:10.3389/fimmu.2023.1071553)
Supplement: Supplementary Table II — Diameter, hemodynamics, and rolling velocities of Th1 and Th17cells in the SC at the different phases of EAE. The mean blood flow velocity (Vm), wall shear stress (WSS), and the percentage of rolling and arrested cells were calculated as described in Materials and Methods. At least 100 consecutive cells/venule were examined. The velocity of rolling cells was measured by digital frame-by-frame analysis of videotapes. Vroll are presented as median. Data are arithmetic mean ± SD for hemodynamic parameters and mean ± SEM for the percentages of rolling and arrest. [file Table_2.pdf]

Supplementary Table II.

EAE course		Score	No. animals/ venules	Diameter (μm)	V_{max} (μm/s)	V_m (μm/s)	WSS (dyne/cm^2)	V_{roll} (μm/s)	% Rolling	% Adhesion
**Preclinical phase**	**Th1**	0 ± 0	3/13	24.6 ± 6.8	1701 ± 349	1097 ± 115	9.3 ± 3.9	31.3	26.5 ± 2.8	4.4± 0.8
	**Th17**	0 ± 0	3/13	24.6 ± 6.8	1528 ± 500	1027 ± 415	9.4 ± 6.6	31.1	19.4 ± 1.8	1.4 ± 0.3
**Disease peak**	**Th1**	2.2 ± 0.3	3/14	31.8 ± 10.3	1616 ± 382	882 ± 207	4.8 ± 1.1	33.6	21.0 ± 2.0	2.2 ± 0.3
	**Th17**	2.2 ± 0.3	3/14	31.8 ± 10.3	1099 ± 222	598 ± 113	3.2 ± 0.4	45.6	26.1 ± 2.1	3.8 ± 0.6
**Chronic phase**	**Th1**	2 ± 0	3/16	22.0 ± 6.2	1558 ± 316	1133 ± 206	10.7 ± 5.3	45.8	25.5 ± 1.9	2.3 ± 0.4
	**Th17**	2 ± 0	3/16	22.0 ± 6.2	1654 ± 280	1055 ± 148	9.8 ± 4.1	53.3	20.0 ± 1.8	2.8 ± 0.7
