# Supplementary material for: Alpha4 beta7 integrin controls Th17 cell trafficking in the spinal cord leptomeninges during experimental autoimmune encephalomyelitis
Source: Front Immunol. 2023 Apr 18;14:1071553. doi: 10.3389/fimmu.2023.1071553 (PMC10151683; doi:10.3389/fimmu.2023.1071553)
Supplement: Supplementary Table III — Diameter, hemodynamics, and rolling velocities of Th1 cells treated with blocking antibodies. Vm, WSS, and the percentage of rolling and arrested cells were calculated as described in Materials and Methods. At least 100 consecutive cells/venule were examined. The velocity of rolling cells was measured by digital frame-by-frame analysis of videotapes. Rolling velocity (Vroll) are presented as median. Data are arithmetic mean ± SD for hemodynamic parameters and mean ± SEM for the percentages of rolling and arrest. [file Table_3.pdf]

Supplementary Table III.

EAE course		Score	No. animals/ venules	Diameter (μm)	V_{max} (μm/s)	V_m (μm/s)	WSS (dyne/cm^2)	V_{roll} (μm/s)	% Rolling	% Adhesion
**Preclinical phase**	**CTR**	0 ± 0	3/13	24.1 ± 6.2	1157 ± 251	787 ± 115	7.5 ± 1.9	53.7	28.5 ± 3.8	6.8 ± 1.4
	**anti-α4**	0 ± 0	3/13	24.1 ± 6.2	1183 ± 294	802 ± 96	7.6 ± 1.6	81.8	12.2 ± 4.0	2.5 ± 0.8
	**CTR**	0 ± 0	3/18	18.1 ± 3.1	2550 ± 1548	1804 ± 996	18.3 ± 9.3	27.4	31.4 ± 8.0	4.3 ± 1.7
	**anti-LFA-1**	0 ± 0	3/18	18.1 ± 3.1	2798 ± 1481	2005 ± 966	20.6 ± 9.4	42.3	12.3 ± 3.0	0.6 ± 0.6
**Disease peak**	**CTR**	2.3 ± 0.6	3/14	32.0 ± 10.5	1694 ± 948	950 ± 474	5.5 ± 2.7	58.3	10.2 ± 2.5	1.4 ± 0.6
	**anti-α4**	2.3 ± 0.6	3/14	32.0 ± 10.5	2251 ± 924	1258 ± 416	7.1 ± 2.4	74.3	3.4 ± 1.8	0.2 ± 0.2
	**CTR**	2 ± 0	3/15	26.8 ± 14.2	1365 ± 264	927 ± 171	9.0 ±1.6	38.4	34.1 ± 5.8	6.3 ± 0.9
	**anti-α4β7**	2 ± 0	3/15	26.8 ± 14.2	1585 ± 251	1078 ± 178	10.4 ± 1.9	42.6	29.1 ± 3.6	6.0 ± 0.5
	**CTR**	2.5 ± 0	3/19	25.2 ± 7.2	1348 ± 153	785 ± 91	5.5 ± 0.9	31.4	25.9 ± 2.7	2.1 ± 0.5
	**anti-LFA-1**	2.5 ± 0	3/19	25.2 ± 7.2	1600 ± 180	933 ± 115	6.5 ± 1.3	28.3	15.1 ± 2.2	1.0 ± 0.3
**Chronic phase**	**CTR**	1.7 ± 0.6	3/16	21.9 ± 6.3	1785 ± 876	1030 ± 462	7.0 ± 2.4	47.6	17.3 ± 4.8	2.1 ± 1.1
	**anti-α4**	1.7 ± 0.6	3/16	21.9 ± 6.3	2094 ± 1091	1206 ± 579	8.2 ± 3.0	53.2	11.3 ± 1.8	0.0 ± 0.0
	**CTR**	2 ± 0	3/12	23.9 ± 2.6	1088 ± 114	799 ± 160	8.1 ± 3.2	48.7	24.6 ± 5.5	4.6 ± 1.9
	**anti-α4β7**	2 ± 0	3/12	23.9 ± 2.6	1269 ± 79	917 ± 143	9.1 ± 3.3	51.2	23.0 ± 1.5	4.6 ± 3.3
	**CTR**	2.2 ± 0.3	3/15	22.7 ± 8.2	1757 ± 401	1213 ± 535	11.6 ± 8.9	49.0	22.4 ± 3.1	1.5 ± 0.6
	**anti-LFA-1**	2.2 ± 0.3	3/15	22.7 ± 8.2	1477 ± 543	1025 ± 527	9.9 ± 7.8	50.3	19.6 ± 2.3	1.5 ± 0.5
