# Supplementary material for: Alpha4 beta7 integrin controls Th17 cell trafficking in the spinal cord leptomeninges during experimental autoimmune encephalomyelitis
Source: Front Immunol. 2023 Apr 18;14:1071553. doi: 10.3389/fimmu.2023.1071553 (PMC10151683; doi:10.3389/fimmu.2023.1071553)
Supplement: Supplementary Table IV — Diameter, hemodynamics, and rolling velocities of Th17 cells treated with blocking antibodies. Vm, WSS, and the percentage of rolling and arrested cells were calculated as described in Materials and Methods. At least 100 consecutive cells/venule were examined. The velocity of rolling cells was measured by digital frame-by-frame analysis of videotapes. Rolling velocity (Vroll) are presented as median. Data are arithmetic mean ± SD for hemodynamic parameters and mean ± SEM for the percentages of rolling and arrest. [file Table_4.pdf]

Supplementary Table IV.

EAE course		Score	No. animals/ venules	Diameter (μm)	V_{max} (μm/s)	V_m (μm/s)	WSS (dyne/cm^2)	V_{roll} (μm/s)	% Rolling	% Adhesion
**Preclinical phase**	**CTR**	0 ± 0	3/13	22.0± 6.3	1474 ± 199	886 ± 93	6.8 ± 0.9	26.0	13.3 ± 1.8	1.6 ± 0.7
	**anti-α4**	0 ± 0	3/13	22.0 ± 6.3	1596 ± 219	962 ±125	7.4 ± 1.4	32.7	11.0 ± 1.2	1.5 ± 0.5
	**CTR**	0 ± 0	3/15	20.4 ± 4.2	1470 ± 441	955 ± 312	8.6 ± 3.4	81.7	19.5 ± 3.6	1.1 ± 0.4
	**anti-LFA-1**	0 ± 0	3/15	20.4 ± 4.2	1557 ± 469	998 ± 272	8.8 ± 2.3	83.0	14.5 ± 4.5	1.3 ± 0.8
**Disease peak**	**CTR**	2 ± 0	3/14	29.2 ± 8.8	1453 ± 740	861 ± 429	6.3± 3.4	45.6	30.4 ± 4.2	1.8 ± 1.4
	**anti-α4**	2 ± 0	3/14	29.2 ± 8.8	1850 ±1044	1095 ± 594	8.0 ± 4.3	41.5	30.8 ± 6.9	0.4 ± 0.4
	**CTR**	2 ± 0	3/24	25.4 ± 7.0	1406 ± 405	894 ± 217	7.2 ± 1.2	29.0	38.7 ± 4.4	5.0 ± 1.3
	**anti-α4β7**	2 ± 0	3/24	25.4 ± 7.0	1409 ± 340	863 ± 174	6.9 ± 0.8	36.6	40.4 ± 2.5	2.4 ± 1.0
	**CTR**	2 ± 0	3/10	28.4 ± 9.1	1746 ± 764	957 ± 426	5.3 ± 2.7	30.7	22.7 ± 4.0	5.5 ± 1.5
	**anti-LFA-1**	2 ± 0	3/10	28.4 ± 9.1	2004 ± 753	1095 ± 413	5.9 ± 2.5	41.9	20.7 ± 3.7	4.1 ± 1.1
**Chronic phase**	**CTR**	2 ± 0	3/16	20.8 ± 6.3	2272 ± 485	1307 ± 226	8.8 ± 1.0	40.5	13.8 ± 1.8	1.4 ± 0.4
	**anti-α4**	2 ± 0	3/16	20.8 ± 6.3	2222 ± 366	1280 ± 160	8.7 ± 1.0	49.4	14.5 ± 3.6	0.7 ± 0.2
	**CTR**	2 ± 0	3/12	21.4 ± 4.1	1454 ± 218	996 ± 273	9.3 ± 4.4	29.0	27.3 ± 4.9	5.0 ± 1.3
	**anti-α4β7**	2 ± 0	3/12	21.4 ± 4.1	1477 ± 272	1019 ± 294	9.7 ± 4.5	30.5	26.4 ± 6.2	0.6 ± 0.5
	**CTR**	1.5 ± 0.7	3/15	21.9 ± 6.0	1598 ± 497	1084 ± 545	10.1 ± 8.4	24.2	24.5 ± 2.8	3.7 ± 1.2
	**anti-LFA-1**	1.5 ± 0.7	3/15	21.9 ± 6.0	1330 ± 573	908 ± 526	8.6 ± 7.4	38.5	23.4 ± 5.7	2.6 ± 0.7
